# Overcoming NADPH product inhibition improves D-sorbitol conversion to L-sorbose

**DOI:** 10.1038/s41598-018-37401-0

**Published:** 2019-01-28

**Authors:** Tae-Su Kim, Hui Gao, Jinglin Li, Vipin C. Kalia, Karthikeyan Muthusamy, Jae Kyung Sohng, In-Won Kim, Jung-Kul Lee

**Affiliations:** 1 Department of Chemical Engineering, Konkuk University, 1 Hwayang-Dong, Gwangjin-Gu, Seoul, 05029 Republic of Korea; 2 Department of Life Science and Biochemical Engineering, SunMoon University, 70 Sunmoon-ro 221, Tangjeong-myeon, Asan-si, Chungnam, 31460 Republic of Korea; 3 Department of Bioinformatics, Alagappa Uiversity, Karaikudi, Tamil Nadu India

**Keywords:** Microbiology techniques, Microbiology techniques, Metabolic engineering, Metabolic engineering

## Abstract

*Gluconobacter oxydans* sorbitol dehydrogenase (GoSLDH) exhibits a higher catalytic efficiency than other l-sorbose producing enzymes. During the reaction catalysed by GoSLDH, NADP^+^ is reduced to NADPH and d-sorbitol is oxidized to l-sorbose. However, GoSLDH activity is inhibited by the NADPH (*K*_i_ = 100 μM) formed during the enzymatic reaction. Therefore, *Escherichia coli*_*gosldh-lrenox*_ producing both GoSLDH for d-sorbitol oxidation and LreNOX (NAD(P)H oxidase from *Lactobacillus reuteri*) for NADP^+^ regeneration was generated and used for l-sorbose production. Whole cell biocatalysts with the LreNOX cofactor recycling system showed a high conversion rate (92%) of d-sorbitol to l-sorbose in the presence of low concentration of NADP^+^ (0.5 mM). By alleviating NADPH accumulation during the catalytic reactions, *E*. *coli*_*gosldh-lrenox*_ exhibited 23-fold higher conversion rate of d-sorbitol than *E*. *coli*_*gosldh*_. l-Sorbose production by *E*. *coli*_*gosldh-lrenox*_ reached 4.1 g/L after 40 min, which was 20.5-fold higher than that of *E*. *coli*_*gosldh*_. We also constructed *G*. *oxydans*_*gosldh*_ and *G*. *oxydans*_*gosldh-lrenox*_ strains, and they exhibited 1.2- and 2.9-fold higher conversion rates than the wild-type *G*. *oxydans* KCTC 1091. The results indicate that overcoming NADPH product inhibition using LreNOX improves chemical production in NADP^+^-dependent enzymatic reactions.

## Introduction

Rare sugars are among the most widely used chemicals in the food and pharmaceutical industries, as building blocks for anticancer and antiviral drugs^1^. These molecules also stimulate the human immune system and are used to control diabetes^2,3^. One of most important rare sugars is L-sorbose. l-Sorbose is largely used as a starting material for l-ascorbic acid biosynthesis^4,5^. It has been also used to synthesize the potent glycosidase inhibitor 1-deoxygalactonojirim^6^ and rare sugars such as l-tagatose^7^ and l-iditol^8^.

Rare sugars can be produced using NAD(P)^+^-dependent polyol dehydrogenases. Steady-state kinetic analysis of polyol dehydrogenases revealed that the dissociation of NAD(P)H is the main rate-limiting step under substrate-saturated reaction conditions^9^. As demonstrated previously, however, polyol dehydrogenases, such as d-sorbitol dehydrogenase^10,11^, l-glutamate dehydrogenase^12^, and mannitol dehydrogenase^13,14^ suffer from product inhibition by NAD(P)H in a competitive manner^9^, revealing a rate-limiting step after the binding of NAD(P)H. NAD(P)H product inhibition must be overcome for rare sugar production using polyol dehydrogenases.

The most widely used industrial method for producing l-sorbose is the biotransformation of d-sorbitol to l-sorbose using *Gluconobacter* or *Acetobacter* species^15,16^. In our previous study, GoSLDH showed the higher activity towards d-sorbitol compared to other polyol dehydrogenases^17^. During the reaction catalysed by GoSLDH, d-sorbitol is oxidized to l-sorbose, which involves the reduction of NADP^+^ to NADPH. Thus, during bioconversion, NADP^+^ is depleted while NADPH and l-sorbose are accumulated. However, NADPH accumulation likely inhibits GoSLDH activity. Therefore, incorporating a cofactor NADP^+^ regeneration system is necessary to release cofactor NADPH inhibition and obtain high l-sorbose productivity in the presence of low concentrations of NADP^+^. Additionally, because of the high cost of pyridine cofactors, an efficient cofactor regeneration system is a prerequisite for the commercial viability of this process^18–20^. Whole cells contain NAD^+^ and NADP^+^ reservoirs that provide a continuous source of cofactors^21^. Therefore, whole cells are used in many applications involving dehydrogenases. Simultaneous overexpression of the target enzymes and cofactor regeneration biocatalyst has been implemented in many asymmetric reduction systems^22,23^.

In the current study, we characterized a novel water-forming LreNOX from *L*. *reuteri* showing a high cofactor preference towards NADPH unlike most other NAD(P)H oxidases (NOXs) which exclusively use NADH as a substrate. We developed a co-expression system in which GoSLDH encoded by the *G*. *oxydans* G624 *gosldh* was used as the l-sorbose producing enzyme and LreNOX was used as the cofactor-regenerating enzyme. Further, we demonstrated a simple, highly efficient, and economical whole-cell biocatalysis system comprised of GoSLDH coupled with LreNOX to regenerate the cofactor NADP^+^ from NADPH. This system reduces the NADPH inhibition effect in the GoSLDH reaction and enables high production of l-sorbose from d-sorbitol.

## Results and Discussion

### GoSLDH inhibition by NADPH

**Figure 1:**
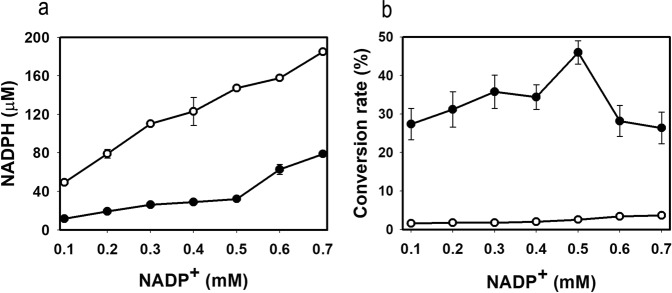
(**a**) NADPH accumulation and (**b**) l-sorbose conversion rate. NADPH concentration and l-sorbose conversion rates in whole cell biocatalysis in the presence of various NADP^+^ concentrations were obtained using whole cells expressing GoSLDH with (opened circle) or without (filled circle) LreNOX co-expression. Data are for reactions in 100 mM glycine-NaOH buffer, pH 10, 50 mM d-sorbitol.

**Figure 2:**
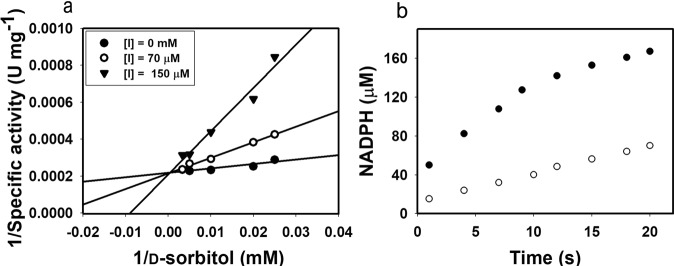
(**a**) Graphical analysis of the inhibition of GoSLDH by NADPH. Analysis of these data by double-reciprocal plots indicated that NADPH inhibited GoSLDH competitively. (**b**) NADPH accumulation during GoSLDH reaction in the presence of 0.5 mM NADP^+^ as a coenzyme. NADPH concentration was obtained using whole cells expressing GoSLDH with (opened circle) or without (filled circle) LreNOX co-expression. Data are for reactions in 100 mM glycine-NaOH buffer, pH 10, 200 mM d-sorbitol.

*Escherichia coli*_pET28-*gosldh*_ carrying GoSLDH, which encodes SLDH from *G*. *oxydans* G624, was constructed in our previous study^17^. During the catalytic process, NADP^+^ was reduced to NADPH, and d-sorbitol was oxidized to l-sorbose. Purified GoSLDH from the induced *E*. *coli*_pET28-*gosldh*_ showed high catalytic activity of 3570 U mg protein^−1^ (in the direction of l-sorbose production). During whole cell biocatalysis by *E*. *coli*_*gosldh*_ in the presence of 0.5 mM NADP^+^, the NADPH concentration reached 147 μM, showing a conversion rate of d-sorbitol to l-sorbose of only 2.6% (Fig. 1a,b). To gain insight into the inhibitory effects of NADPH, the inhibition constant (*K*_i_) of NADPH for GoSLDH was determined. Double reciprocal plots revealed competitive inhibition of GoSLDH by NADPH (Fig. 2a). The *K*_i_ value of NADPH was determined to be 100 µM. Inhibition kinetic analysis, performed with NADPH concentrations ranging from 0 to 170 µM, showed that the apparent *K*_m_ increased as the NADPH concentration increased. *K*_m_ increased from 3.9- to 8.9-fold compared to the values without supplementary NADPH as NADPH concentration increased from 70 to 150 µM. After 20 s of *E*. *coli*_*gosldh*_ whole cell biocatalysis, up to 160 μM NADPH was accumulated, resulting in 80% inhibition of GoSLDH activity (Fig. 1b). The reduction in NADPH formation would likely increase l-sorbose production by reducing GoSLDH inhibition. Therefore, we established a cofactor regeneration system using a low initial concentration (0.5 mM) of NADP^+^.

### Binding thermodynamics studies

**Figure 3:**
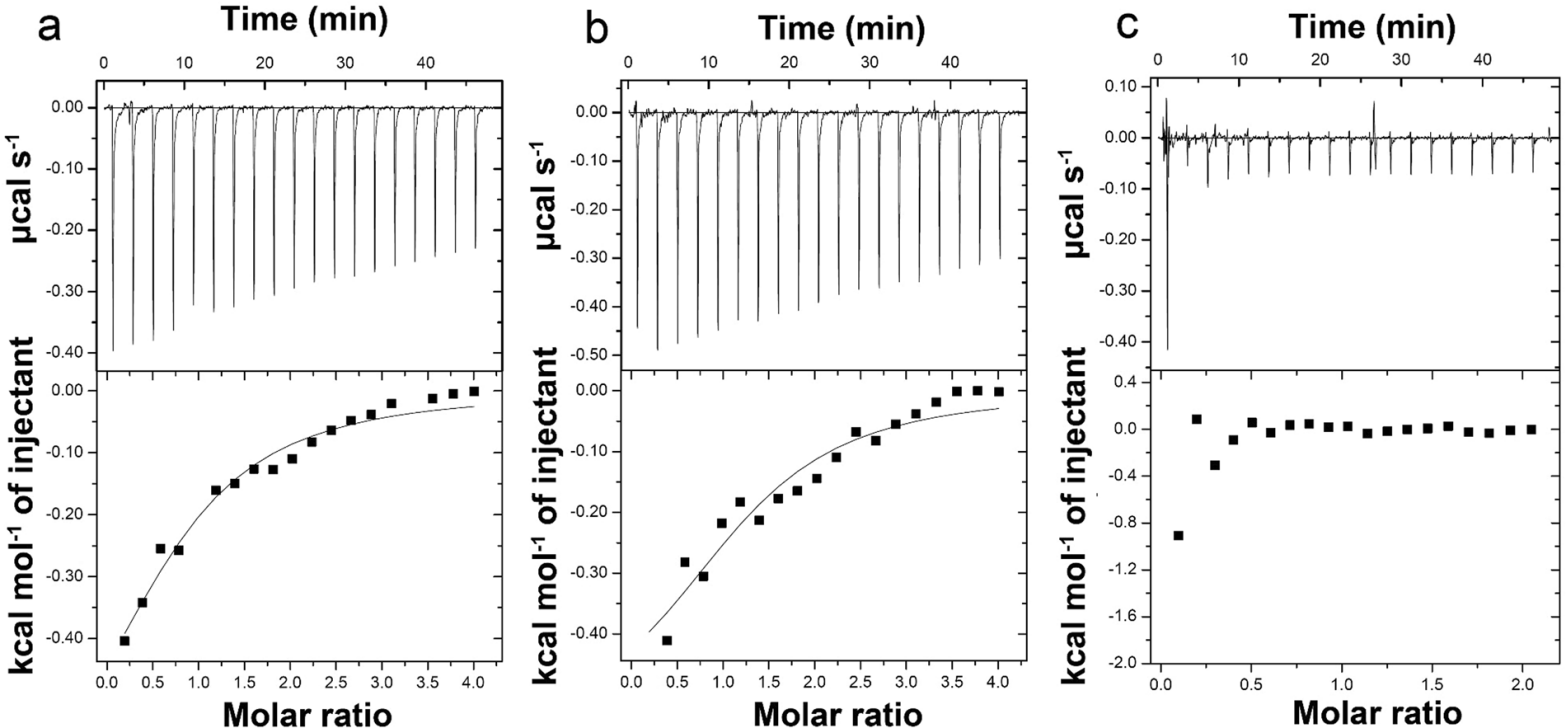
Representative ITC results and fitting curves for (**a**) NADP^+^ binding to GoSLDH (100 μM); (**b**) NADPH binding to GoSLDH (100 μM) in the absence of NADP^+^; and (**c**) NADPH binding to GoSLDH (100 μM) in the presence of NADP^+^ (100 μM). ITC experiments were performed to characterize the binding thermodynamics of NADP^+^ to GoSLDH (100 μM) in 100 mM glycine-NaOH buffer (pH 10) at 25 °C.

We performed isothermal titration calorimetry (ITC) experiments to characterize the binding thermodynamics of NADP^+^ to GoSLDH (100 μM) in 100 mM glycine-NaOH buffer (pH 10) at 25 °C. As shown in Fig. 3a, the binding of NADP^+^ to GoSLDH in the absence of d-sorbitol was exothermic (ΔH =-754 cal mol^−1^) with a K_a_ value of 1.12 × 10^4^ M^−1^ or K_d_ (dissociation constant) value of 89.3 μM. This is equal to a ΔG value of −5.5 kJ mol^−1^, showing that the binding is mostly entropy-driven (with a calculated ΔS of 16.0 cal mol^−1^ deg^−1^). Next, binding of NADPH to GoSLDH (100 μM) was found to be exothermic (ΔH = −578 cal mol^−1^) with K_a_ (affinity constant) value of 1.49 × 10^4^ M^−1^ or a K_d_ value of 67.1 μM, indicating the higher binding affinity of GoSLDH for NADP^+^ (22 μM lower K_d_) (Fig. 3b). This equals a ΔG value of −5.6 kJ mol^−1^, showing the binding is mostly entropy-driven (with a calculated ΔS of 17.1 cal mol^−1^ deg^−1^). NADP^+^ was also titrated into GoSLDH (100 μM) in the presence of NADPH (300 μM). As shown in Fig. 3c, binding of NADP^+^ to the protein was not detected, suggesting that NADPH and NADP^+^ compete with each other because these two molecules bind to the same site in GoSLDH. Because GoSLDH does not reduce reaction activity at 25 °C in the presence of 200 mM l-sorbose and 0.5 mM NADPH, NADPH solely inhibits the oxidation reaction of GoSLDH.

### Characterization of NADH oxidase

NOXs catalyse the oxidation of NAD(P)H by simultaneously reducing molecular O_2_ to water or hydrogen peroxide^24–26^. NOXs play important roles in regenerating oxidized cofactors required for numerous enzymatic reactions. Because nearly all proteins are deactivated upon exposure to H_2_O_2_, H_2_O-forming NOXs are considered as more effective than H_2_O_2_-forming NOXs for NAD(P)^+^ regeneration^24^. Most NOXs show NADH oxidation activity rather than NADPH oxidation activity. In this study, we characterized a novel NOX from *L*. *reuteri* (LreNOX) which efficiently oxidized both NADPH and NADH. The NOX gene from *L*. *reuteri* was isolated from genomic DNA using specific primers derived from the coding sequence (Table 1). The gene was cloned into the T7 promoter-based plasmid pET28a to produce pET28a-lrenox and then heterologously expressed in *E*. *coli* BL21 (DE3) cells. The enzyme was purified by nickel–nitrilotriacetic acid affinity chromatography; purity was assessed by polyacrylamide gel electrophoresis in the presence of sodium dodecyl sulphate (Fig. S1). The optimal pH and temperature for the oxidation of NADPH by purified LreNOX were 6.0 and 60 °C, respectively. Investigation of the kinetic parameters with NADPH as a substrate was performed using the purified enzyme LreNOX under the optimal assay conditions. Figure S2 shows that LreNOX oxidized NADPH with a high specific activity (*V*_max_ = 161 U mg^−1^) and binding affinity (*K*_m_ = 35 µM). Compared to *L*. *san*-Nox2, LreNOX showed 14.6-fold higher activity towards NADPH. This suggests that LreNOX is a good candidate for enzymatic regeneration of the oxidized cofactor NADP^+^.

**Table 1:** Bacterial strains, plasmids, and primers used in this study.

Strain, plasmids, or primers	Genotype, properties, or sequence	Source
Bacteria		
*E. coli* DH5α	F^−^, ϕ80d/*lac*ZΔM15, Δ(*lac*ZYA-*arg*F)U169, *deo*R, *rec*A1, *end*A1, *hsd*R17(rk^−^mk^+^,), *pho*A, *sup*E44, λ^−^, thi^−1^, *gyr*A96, *rel*A1	Clontech
*E. coli* BL21–Codon Plus (DE3)-RIL	B F *ompT hsdS*(r_B_ m_B_^-^) *dcm*^+^ Tc^r^ *gal* (DE3) *endA* The [*argU ileY leuW* Cam^r^]	Stratagene
*E. coli*_pET28- *gosldh*_	Km^r^, *gosldh* in pET28a in *E. coli* BL21-Codon Plus (DE3)-RIL	20
*E. coli*_pET28- *lrenox*_	Km ^r^, *lrenox* in pET28a in *E. coli* BL21–Codon Plus (DE3)-RIL	This study
*E. coli*_gosldh- *lrenox*_	*gosldh* and *lrenox* in pETDuet-1 vector in *E. coli* BL21–Codon Plus (DE3)-RIL	This study
*E. coli*_*gosldh*_	*Gosldh* in pETDuet-1 vector in *E. coli* BL21-Codon Plus (DE3)-RIL	This study
Plasmids		
pET28a	Km^r^, overexpression vector	Novagen
pETDuet-1	Amp^r^, overexpression vector	Novagen
pGEM	Amp^r^, cloning vector	Promega
pGEM-T-nox	Amp^r^, Lrenox in T-vector	This study
pETDuet-*gosldh*	Amp^r^, *gosldh* in pETDuet	This study
Primers	Sequence (5′-s3′)	Restriction enzyme site
Nox1	AAA GGA TCC AAT GAA GGT TAT TAT TGT T	*BamHI*
Nox2	GTG GCG GCC GCT TAT TTT TCT AAT TCA GC	*NotI*
PadhF	GTGAGGTACCTCCCGCCCGGTTTCG	
PadhR	AGGGTTTCGCGCGTAATCATGATCCAACTGTCCTTTTTGT	
gosldhF	ACAAAAAGGACAGTTGGATCATGATTACGCGCGAAACCCT	
gosldhR	TGACTCGAGTCAGGCCGGGATGGCGG	
PadhLF	GTGAAAGCTTTCCCGCCCGGTTTCG	
padhLR	CCGACGATGATGACCTTCATGATCCAACTGTCCTTTTTGT	
lrenoxF	ACAAAAAGGACAGTTGGATCATGAAGGTCATCATCGTCGG	
lrenoxR	GATGGATCCTCACTTTTCCAGTTCGG	

### Strategy for overcoming NADPH inhibition in l-sorbose production

GoSLDH catalyses strict NADP^+^-dependent conversion of d-sorbitol to l-sorbose and exhibits NADPH product inhibition. Accumulation of NADPH during the SLDH reaction is a bottleneck in the conversion of d-sorbitol to l-sorbose. Therefore, it is important to reduce NADPH accumulation to increase l-sorbose production. To make the reaction more efficient and economical, it is necessary to regenerate the cofactor NADP^+^ from NADPH. In the present study, we used highly active LreNOX to regenerate NADP^+^. In the coupled system of GoSLDH and LreNOX, LreNOX recycled the reduced cofactor NADPH in the reaction mixture to the oxidized cofactor NADP^+^. Cofactor regeneration may provide an alternative tool for not only reducing the product NADPH concentration but also minimizing the reaction cost because of the lower concentration (0.5 mM NADP^+^) of the initial cofactor (Fig. 1a).

When we used *E*. *coli* BL21-Codon Plus (DE3)-RIL cells, no conversion of d-sorbitol to l-sorbose was observed. Therefore, we used *E*. *coli* BL21-Codon Plus (DE3)-RIL cells as an expression host for *lrenox* and *gosldh*. The biocatalytic activities of the purified enzymes prepared from *E*. *coli*_pET28-*lrenox*_ and *E*. *coli*_pET28-*gosldh*_ cells were assayed using d-sorbitol as a substrate. First, the enzyme was purified from induced *E*. *coli*_pET28-*gosldh*_ (Table 1). The purified GoSLDH showed a high SLDH activity of 3570 U mg^−1^ in the direction of l-sorbose production. Second, *E*. *coli*_pet28*-lrenox*_ harbouring *lrenox*, which encodes LreNOX from *L*. *reuteri*, was constructed. Most recombinant wild-type NOXs showed activity with NADH, but less or no activity with NADPH. To regenerate NADP^+^ for l-sorbose production, we screened NOXs from different organisms and found that LreNOX can oxidise NADPH. LreNOX exhibited higher activity towards NADPH (160 U mg^−1^) than previously reported NOXs.

**Figure 4:**
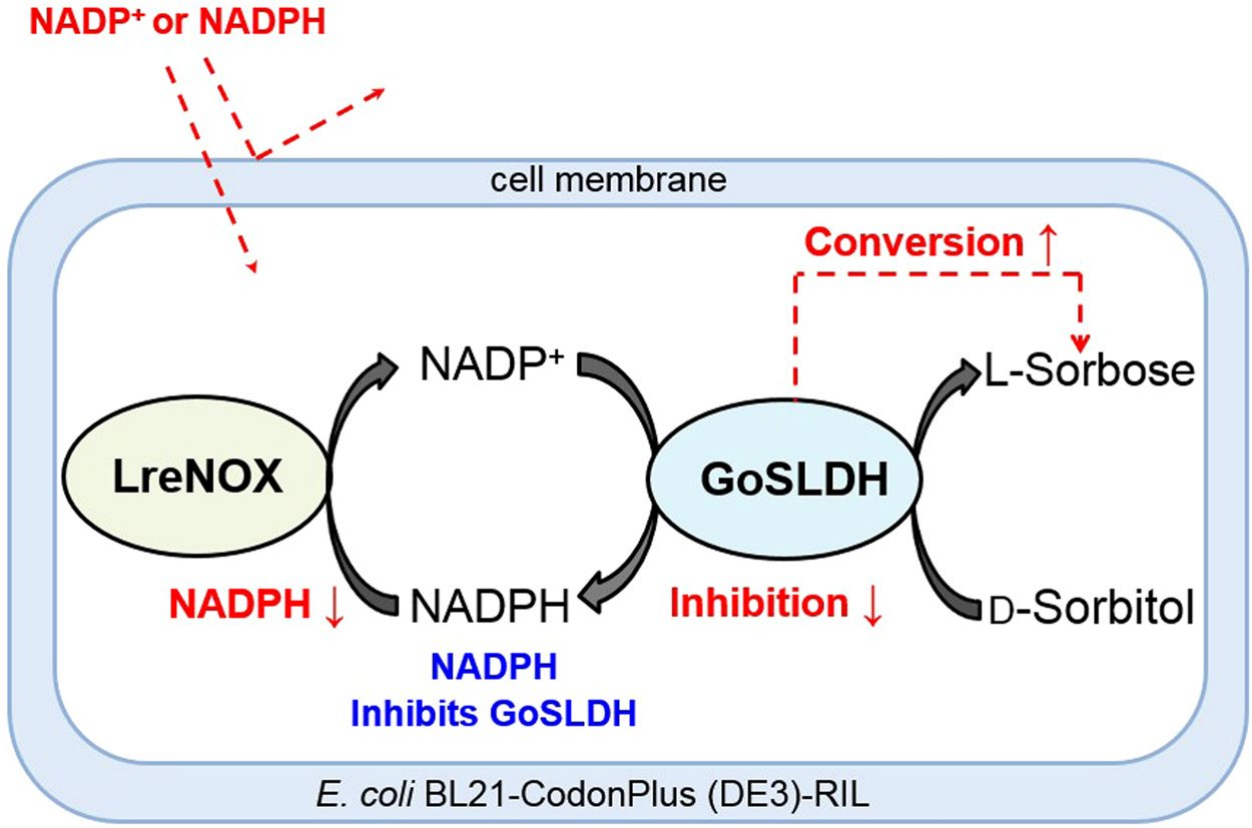
Schematic illustration of biocatalytic l-sorbose production using *Escherichia coli* whole cells harbouring GoSLDH coupled with LreNOX, a cofactor regeneration enzyme.

Because whole cells contain NAD^+^ and NADP^+^ reservoirs as a continuous source of cofactors^21^, *E*. *coli*_pET28-*lrenox*_ was used for cofactor NADP^+^ regeneration in the current study. When GoSLDH and LreNOX are expressed individually in two different cells, the cellular membranes of the cells may limit the exchange of pyridine cofactors between the two cells^27^. To avoid retardation of cofactor exchange, *E*. *coli*_*gosldh-lrenox*_ cells co-expressing LreNOX and GoSLDH were constructed as shown in Fig. 4. LreNOX oxidized the NADPH produced by GoSLDH and provided the oxidized form the cofactor NADP^+^ for the biocatalytic process of GoSLDH, without the limitation of a transmembrane process. Efficient production of l-sorbose was possible by removing NADPH inhibition using *E*. *coli*_*gosldh-lrenox*_ as the biocatalyst and d-sorbitol as the substrate (Fig. 4).

### Effects of pH and cofactor concentration on l-sorbose production

**Figure 5:**
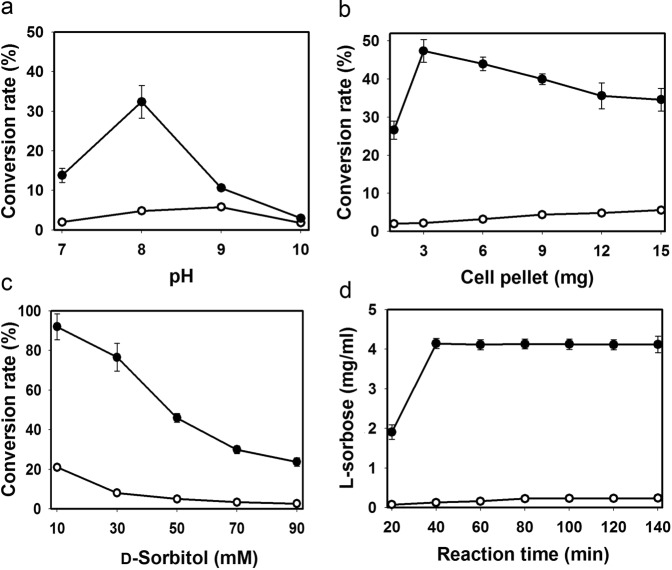
Effects of biocatalysis conditions on the conversion rate. (**a**) pH; (**b**) amount of dry cell pellet (mg); (**c**) d-sorbitol concentration (mM); (**d**) reaction time (min). *E*. *coli*_*gosldh-lrenox*_ and *E*. *coli*_*gosldh*_ were incubated with d-sorbitol and NADP^+^ in the reaction. NADPH concentration and l-sorbose conversion rates were obtained using whole cells expressing GoSLDH with (filled circle) or without (opened circle) LreNOX co-expression.

To achieve high product conversion, the effect of pH on l-sorbose yield from d-sorbitol was investigated. The reaction used 50 mM of d-sorbitol as the initial substrate and 3.0 mg dry cell weight (DCW) mL^−1^ of *E*. *coli*_*gosldh*_ or *E*. *coli*_*gosldh-lrenox*_ as the biocatalyst under different pH conditions. After a 1-h reaction with *E*. *coli*_*gosldh*_ or *E*. *coli*_*gosldh-lrenox*_, the conversion rates were measured by monitoring the product concentration. The conversion rate was highest at pH 8.0 for *E*. *coli*_*gosldh-lrenox*_ (32.4%) and pH 9.0 for *E*. *coli*_*gosldh*_ (5.8%), respectively. The optimal pH values of purified GoSLDH and LreNOX were pH 10.0 and 5.0, respectively (Fig. 5a).

Cofactor concentration is another important parameter in the catalytic process and requires careful monitoring because high cofactor concentrations may lead to enzyme inhibition and high production costs^18,19,28^. The effect of cofactor concentration on l-sorbose production was investigated (Fig. 1). During whole cell biocatalysis with *E*. *coli*_*gosldh*_ in the presence of 0.7 mM NADP^+^, the NADPH concentration reached 147 μM, which is higher than the *K*_i,NADPH_ value (100 μM) of GoSLDH. In contrast, biocatalysis with *E*. *coli*_*gosldh-lrenox*_ produced only 32.2 μM NADPH (Fig. 1a), showing a 42.7% improvement in l-sorbitol conversion. As a result, *E*. *coli*_*gosldh-lrenox*_ exhibited a 17.7-fold increase in d-sorbitol conversion to l-sorbose compared to *E*. *coli*_*gosldh*_ (Fig. 1a). The conversion rates of *E*. *coli*_*gosldh-lrenox*_ and *E*. *coli*_*gosldh*_ were 46% and 3.4%, respectively, under the optimal NADP^+^ concentration. LreNOX co-expression was beneficial for producing l-sorbose because of the reduction in NADPH accumulation (Fig. 1). The optimal initial concentration of NADP^+^ was 0.7 mM for producing l-sorbose in the absence of the NADP^+^ regeneration system. Incorporation of LreNOX lowered the NADP^+^ requirement to 0.05 mM for the conversion of sorbitol to l-sorbose. In the current study, therefore, a 14-fold lower NADP^+^ concentration was used with the NADP^+^ regeneration system.

### Effects of cell density and substrate concentration on l-sorbose production

The effect of *E*. *coli*_*gosldh*_ and *E*. *coli*_*gosldh-lrenox*_ cell density on l-sorbose production was investigated (Fig. 5b). The conversion of d-sorbitol to l-sorbose increased in the range of 1.5–3 mg DCW mL^−1^ (*E*. *coli*_*gosldh-lrenox*_). When the *E*. *coli*_*gosldh-lrenox*_ DCW was increased to greater than 3.0 mg mL^−1^, the production of l-sorbose remained nearly unchanged, indicating that the optimal cell amount was 3.0 mg mL^−1^. Under the optimized conditions, *E*. *coli*_*gosldh-lrenox*_ exhibited a 47.4% conversion rate when we used 50 mM d-sorbitol, 0.5 mM NADP^+^, and 3.0 mg DCW mL^−1^. For *E*. *coli*_*gosldh*_, the conversion rate was only 2–5.6%, although 1.5–15 mg DCW mL^−1^ was used.

Substrate concentration is an important parameter determining the rate of biocatalysis. Therefore, we investigated the effect of substrate concentration on the conversion rate by varying the d-sorbitol concentration over 10–90 mM. The maximum conversion was 92.0% and 23.7% when 10 mM d-sorbitol was used with whole cell biocatalysts *E*. *coli*_*gosldh-lrenox*_ and *E*. *coli*_*gosldh*_, respectively. The maximum l-sorbose concentrations were 4.1 and 0.23 g/L, respectively, when whole cell biocatalysts *E*. *coli*_*gosldh-lrenox*_ and *E*. *coli*_*gosldh*_ were used in presence of 50 mM d-sorbitol. Because of the oxidizing activities of membrane-bound dehydrogenases, *G*. *oxydans* has been used for industrial production of l-sorbose for decades^29–31^. Heterogeneous expression of *sldh* in *Pseudomonas putida* IF03738 was reported previously^32^, and purified SLDH showed 61.5% conversion with 1.12 g/L of l-sorbose at 25 °C for 24 h. In the current study, we obtained 3.7-fold higher production of l-sorbose with the whole cell biocatalyst *E*. *coli*_*gosldh-lrenox*_ than that obtained with the SLDH system expressed in *P*. *putida* IF03738^32^.

### Effects of *lrenox* expression in *G*. *oxydans*

To further confirm the effect of *lrenox* expression on L-sorbose production, recombinant *G*. *oxydans* strains harboring the pBBR-adh-gosldh and pBBR-adh-gosldh/lrenox plasmids were constructed (Fig. S3). Gene expression of *gosldh* and *lrenox* in *G*. *oxydans* was confirmed by real time PCR using the appropriate primers (Table S1, Fig. S3). *G*. *oxydans* (KCTC 1091), *G*. *oxydans*_*gosldh*_, and *G*. *oxydans*_*gosldh-lrenox*_ showed conversion rates of 33.3%, 40.0%, and 96.6%, respectively, after a 12 h reaction (Fig. S4). The conversion rates of *G*. *oxydans*_*gosldh*_ and *G*. *oxydans*_*gosldh-lrenox*_ were 1.2 and 2.9 times higher, respectively, than that of wild-type *G*. *oxydans*. We also compared productivity of the strains. The L-sorbose productivity of *G*. *oxydans*_*gosldh-lrenox*_ (145 g/L/h of L-sorbose from 150 g/L of D-sorbitol during a 12 h reaction) was 2.5 and 2.4 times higher than that of *G*. *oxydans* KCTC 1091 and *G*. *oxydans*_*gosldh*_ strains, respectively. Results suggest that enhanced productivity of the *G*. *oxydans*_*gosldh-lrenox*_ strain is attributable to NADP^+^ regeneration, leading to minimization of NADPH-mediated GoSLDH inhibition. We have compared the present conversion rate achieved by *E*. *coli* with those achieved by *Gluconobacter* species (Table 2). Although we did not optimize the process for *G*. *oxydans*_*gosldh-lrenox*_ culture in this study, the production rate of L-sorbose (12.1 g/L/h) was similar to the highest level reported previously (13.6 g/L/h). In this manuscript, we report for the first time that NADPH inhibits GoSLDH, and *lrenox* expression minimizes NADPH inhibition, thereby enhancing L-sorbose production by *E*. *coli* and *G*. *oxydans*.

**Table 2:** Conversion rate and productivity of L-sorbose by microorganisms.

Strains	Productivity (g/L/h)	Substrate (g/L)	Conversion rate (%)	Type of strain	Source
*G*. *oxydans* WSH-003	4.0	150	96.6	Wild type	^37^
*G*. *oxydans* WSH-003	5.62	150	90	Recombinant (*sldh*)	^29^
*G*. *oxydans*	13.6	200	100	Mutant	^30^
*G*. *oxydans* WSH-003	9.03	150	96.3	Recombinant (*sldh* with strong promoter)	^34^
*E. coli*	0	0	0	Wild type	This study
*E. coli*	0.30	5.5	3.46	Recombinant (*gosldh*)	This study
*E. coli*	6.15	5.5	78.5	Recombinant (*gosldh* and *lrenox*)	This study
*G*. *oxydans* KCTC 1091	4.79	150	76.6	Wild type	This study
*G*. *oxydans* KCTC 1091	5.04	150	80.7	Recombinant (*gosldh*)	This study
*G*. *oxydans* KCTC 1091	12.1	150	96.6	Recombinant (*gosldh* and *lrenox*)	This study

## Conclusions

GoSLDH catalyses strict NADP^+^-dependent interconversion between d-sorbitol and l-sorbose but shows NADPH product inhibition. To avoid inhibition by NADPH, we coupled a cofactor regenerating LreNOX with GoSLDH to perform bioconversion using whole cells of both *E*. *coli*_*gosldh-lrenox*_ and *E*. *coli*_*gosldh*_ as biocatalysts. In the coupled system, LreNOX was used to remove the reduced cofactor NADPH from the reaction mixture and recycle it to the oxidized cofactor NADP^+^. Biocatalytic synthesis of l-sorbose was successfully performed using the *E*. *coli*_*gosldh-lrenox*_ whole-cell system. Under optimal conditions, the maximum l-sorbose conversion with *E*. *coli*_*gosldh-lrenox*_ reached 92%, which is much higher than that obtained using the SLDH heterogeneous expression system (61.5%)^32^. l-Sorbose production with *E*. *coli*_*gosldh-lrenox*_ was 4.1 g/L after a 40-min reaction, which was 20.5-fold higher than that using *E*. *coli*_*gosldh*_. Strategy for cofactor regeneration provides an efficient tool for preventing NADPH accumulation and product inhibition, and provides a new biocatalytic method for chemical production using the NADP^+^-dependent enzymatic reaction. Additionally, production costs can be reduced because of the lower NADP^+^ amounts required.

## Materials and Methods

### Plasmids and reagents

Ex-Taq DNA polymerase, a genomic DNA extraction kit, pGEM-T easy vector, and reagents for polymerase chain reaction were purchased from Promega (Madison, WI, USA). T4 DNA ligase and restriction enzymes were purchased from New England Biolabs (Ipswich, MA, USA). The pET28a expression vector, plasmid isolation kit, and nickel-nitrilotriacetic acid superflow column for His-tag protein purification were obtained from Qiagen (Hilden, Germany)^20^. Oligonucleotide primers were obtained from Bioneer (Daejeon, Republic of Korea)^29^. Electrophoresis reagents were provided by Bio-Rad (Hercules, CA, USA), and all chemicals used in assays were purchased from Sigma-Aldrich (St. Louis, MO, USA).

### Isothermal titration calorimetry

ITC measurements were performed using an NADP^+^ and NADPH (MicroCal iTC 200 system, GE Healthcare, Little Chalfont, UK) at the Korea Basic Science Institute (Ochang, Republic of Korea). ITC measurements were performed at 25 °C in buffer containing 100 mM glycine-NaOH buffer (pH 10) using an Auto-iTC200 Micro-Calorimeter at the Korea Basic Science Institute^33^. For ITC measurements, of 134 μM GoSLDH was titrated into a calorimeter cell containing 2.6 mM NADP and NADPH with 2-mL injections. The ITC data were analysed using MicroCal Origin^TM^ software^32^.

### Constructions of *E. coli*_*gosldh*_ and *E. coli*_*gosldh-lrenox*_

The synthesized *sldh* sequence was based on the DNA sequence of the Polyol specific long-chain dehydrogenase from *G*. *oxydans G624* (GenBank accession number AB028937.1). Codon optimization was performed as previously reported^17^. The optimized *sldh* sequences were named as *gosldh*. The *gosldh* fragments, flanked by *Nde*I and *Xho*I restriction enzymes sites at the 5′ and 3′ ends, respectively, were synthesized by GeneScript (Piscataway, NJ, USA) and cloned into the pUC57 vector. Codon-optimized *gosldh* was digested by *Nde*I-*Xho*I and then ligated into the expression vector pETDuet-1 from Novagen (Madison, WI, USA) to express *gosldh*. Additionally, the *lrenox* fragment was obtained from the genome of *L*. *reuteri* using primers Nox1 and Nox2 (Table 1). To construct a co-expression system carrying *gosldh* and *lrenox*, pEasy-Blunt-nox was digested with *Bam*HI and *Not*I, and the gel-purified *nox* fragment was ligated to pETDuet-*gosldh* digested with the same restriction enzymes. *Escherichia coli* DH5a cells were used for general cloning, and *E*. *coli* BL21-Codon Plus (DE3)-RIL cells were used for protein expression. Luria-Bertani medium was used for both *E*. *coli*.

### Production of l-sorbose from D-sorbitol by recombinant *E*. *coli*

Recombinant *E*. *coli* cells were grown at 37 °C on a rotary shaker (200 rpm) in Luria-Bertani medium containing ampicillin (100 µg mL^−1^) and chloramphenicol (50 µg mL^−1^). Recombinant gene expression was induced by adding 0.2 mM IPTG at 16 °C to avoid the formation of inactive inclusion bodies. After induction, the cells were harvested by centrifugation at 4,000 rpm for 12 min at 4 °C and then washed twice with distilled water. The cell pellet was resuspended in 100 mM Tris-HCl (pH 7–8) or Glycine-NaOH buffer (pH 9–10) and maintained at 4 °C for further studies. To optimize the parameters of pH and cell density to produce l-sorbose from *E*. *coli*_*gosldh*_, the cell pellets described above were resuspended in 100 mM Tris-HCl buffer (pH 7–8) or Glycine-NaOH buffer of varying pH (9–10), at a cell density of 1.5–18 mg DCW mL^−1^. The effect of d-sorbitol and NADP^+^ concentrations on the rate of l-sorbose production from *E*. *coli*_*gosldh*_ was evaluated by varying the initial d-sorbitol concentration from 10 to 90 mM and NADP^+^ concentration from 0.1 to 0.8 mM. The reaction conditions for the coupled system were the same as those for producing l-sorbose with whole-cell *E*. *coli*_*gosldh*_ alone. *Escherichia coli* carrying the expression plasmid pACYCDuet-1 with the *gosldh* genes was used as the control strain. For all combinations of pH, NADP^+^ concentration, cell density, and substrate concentration, biotransformation of d-sorbitol to l-sorbose was conducted at 30 °C with shaking at 150 rpm. Samples of the cell suspension (1 mL) were obtained periodically and centrifuged at 13,000 rpm for 5 min; the supernatants were used to analyse l-sorbose concentration.

### Overexpression of genes in *G*. *oxydans* KCTC 1091

*G*. *oxydans* KCTC 1091 was purchased from the Korean Collection for Type Cultures (KCTC) and stored at −80 °C. *G*. *oxydans* strains were cultivated in D-sorbitol medium, at 30 °C, 200 rpm for 24 hr^34^. *E*. *coli* pBBR1MCS-2 was cultivated on Luria-Bertani medium with 50 mg/L of kanamycin, at 37 °C, 200 rpm. The promoter (P_*adh*_) region of *G*. *oxydans* 624H (~220 bp)^35^ was synthesized by Bioneer (Daejeon, South Korea). The promoter (P_*adh*_) region and *gosldh* and *lrenox* genes were amplified via overlap PCR, using primer pairs for *gosldh* (PadhF, PadhR, gosldhF, GoSLDHR) and *lrenox* (PadhLF, padhLR, lrenoxF, lrenoxR), respectively (Table S1). PCR-amplified P_*adh*_-gosldh, P_*adh*_-lrenox fragments were digested and cloned into the *KpnI/XhoI* and *Hind* III*/Bam*H1 sites (respectively) of the broad host vector pBBR1MCS-2, to produce pBBR-gosldh and pBBR-gosldh/lrenox (Fig. S3). All constructed vectors were transformed into *G*. *oxydans* KCTC 1091 by electroporation. Transformants were named *G*. *oxydans*_*gosldh*_ and *G*. *oxydans*_*gosldh-lrenox*_, respectively (Fig. S3).

### Isolation of RNA and RT-PCR

Primers for RT-PCR were synthesized by Bioneer (Daejeon, Republic of Korea) and are shown in Table S1. RNA isolation and RT-PCR were performed as previously described^29^.

### Product analytical methods

Samples were withdrawn at regular time intervals and analysed by the cysteine carbazole sulfuric method and absorbance was measured at 560 nm^36^. The results were further confirmed by high-performance liquid chromatography using an Ultimate 3000 high-pressure liquid chromatography system (Dionex, Sunnyvale, CA, USA) equipped with a Shodex sugar sp0810 column (Showa Denko, K. K., Kawasaki, Japan) and evaporation light scattering detector (ESA6700, Chromachem, Chromaflo Technologies, Ashtabula, OH, USA). Samples were eluted with water at a rate of 1 mL min^−1^ at 80 °C (column temperature). The retention times for d-sorbitol and l-sorbose were 21 and 9.6 min, respectively, under the operating conditions.

## Supplementary information

Supplementary Info
