# Author Correction: Overcoming NADPH product inhibition improves D-sorbitol conversion to L-sorbose

**DOI:** 10.1038/s41598-019-48082-8

**Published:** 2019-08-15

**Authors:** Tae-Su Kim, Hui Gao, Jinglin Li, Vipin C. Kalia, Karthikeyan Muthusamy, Jae Kyung Sohng, In-Won Kim, Jung-Kul Lee

**Affiliations:** 1 Department of Chemical Engineering, Konkuk University, 1 Hwayang-Dong, Gwangjin-Gu, Seoul 05029 Republic of Korea; 2 Department of Life Science and Biochemical Engineering, SunMoon University, 70 Sunmoon-ro 221, Tangjeong-myeon, Asan-si, Chungnam 31460 Republic of Korea; 3 Department of Bioinformatics, Alagappa Uiversity, Karaikudi, Tamil Nadu India

Correction to: *Scientific Reports* 10.1038/s41598-018-37401-0, published online 28 January 2019

The original version of this Article contained a typographical error in the name of author Hui Gao, which was incorrectly given as Gao Hui. This has now been corrected in the PDF and HTML versions of the Article, and in the accompanying Supplementary Information file.

