# Supplementary material for: Overcoming NADPH product inhibition improves D-sorbitol conversion to L-sorbose
Source: Sci Rep. 2019 Jan 28;9:815. doi: 10.1038/s41598-018-37401-0 (PMC6349845; doi:10.1038/s41598-018-37401-0)
Supplement: Supplementary file 1 — Supplementary Info [file 41598_2018_37401_MOESM1_ESM.docx]

***Supplementary Information***

**Overcoming NADPH product inhibition improves D-sorbitol conversion to L-sorbose**

**Tae-Su Kim^1,2,†^, Hui Gao^1,†^, Jinglin Li^1^, Vipin C Kalia^1^, Karthikeyan Muthusamy^3^, Jae Kyung Sohng^2^, In-Won Kim^1^*, Jung-Kul Lee^1^***

^1^Department of Chemical Engineering, Konkuk University, 1 Hwayang-Dong, Gwangjin-Gu, Seoul 05029, Republic of Korea

^2^Department of Life Science and Biochemical Engineering, SunMoon University, 70 Sunmoon-ro 221, Tangjeong-myeon, Asan-si, Chungnam 31460, Republic of Korea

^3^Department of Bioinformatics, Alagappa Uiversity, Karaikudi, Tamil Nadu, India.

^†^: These authors equally contributed to this work.

# ^*^: Author for correspondence. E-mail: jkrhee@konkuk.ac.kr, inwon@konkuk.ac.kr


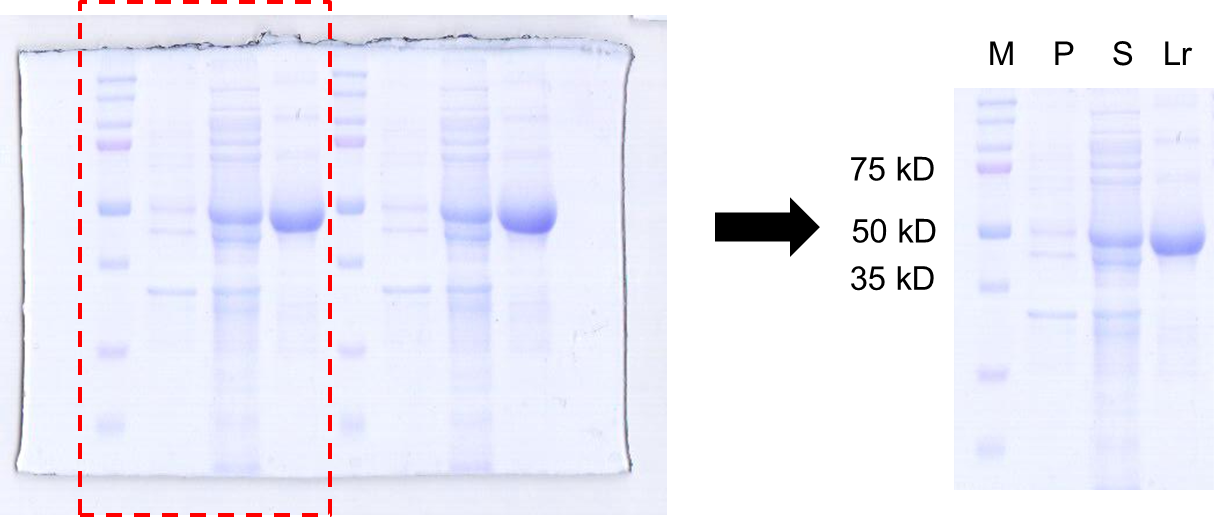


**Figure S1.** Examination of protein expression and purification by SDS–PAGE. Lane M, molecular weight standard marker; lane P, pellet of the cell-free crude extract of *E. coli* BL21 (DE3) harbouring pET28a-LreNOX; lane S, supernatant of the cell-free crude extract of *E. coli* BL21 (DE3) harbouring pET28a-LreNOX; lane Lr, purified LreNOX. Red dotted line displays the cropping area of full-length SDS-PAGE gel.


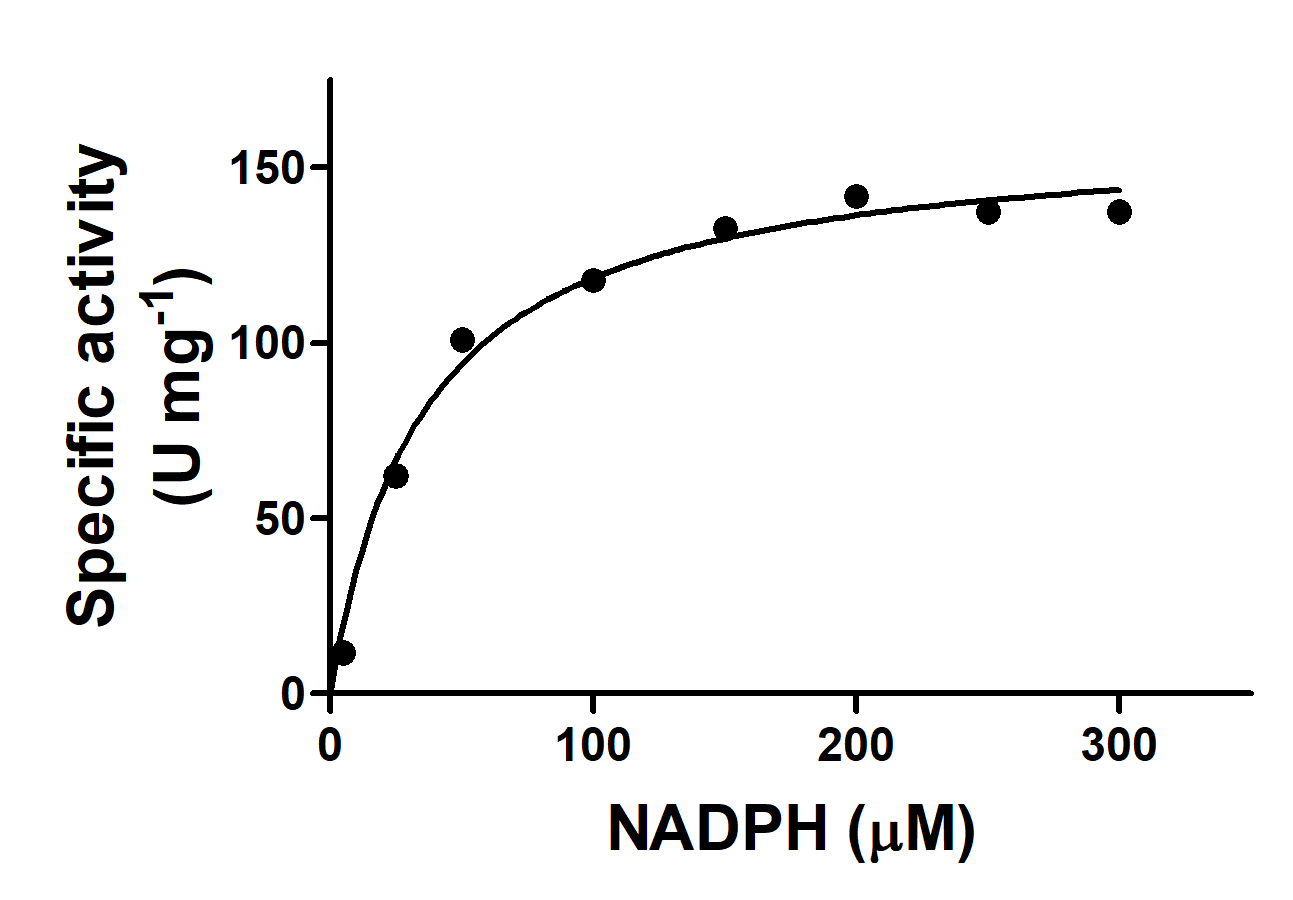


**Figure S2.** Effects of NADPH concentrations on the activity of LreNOX.


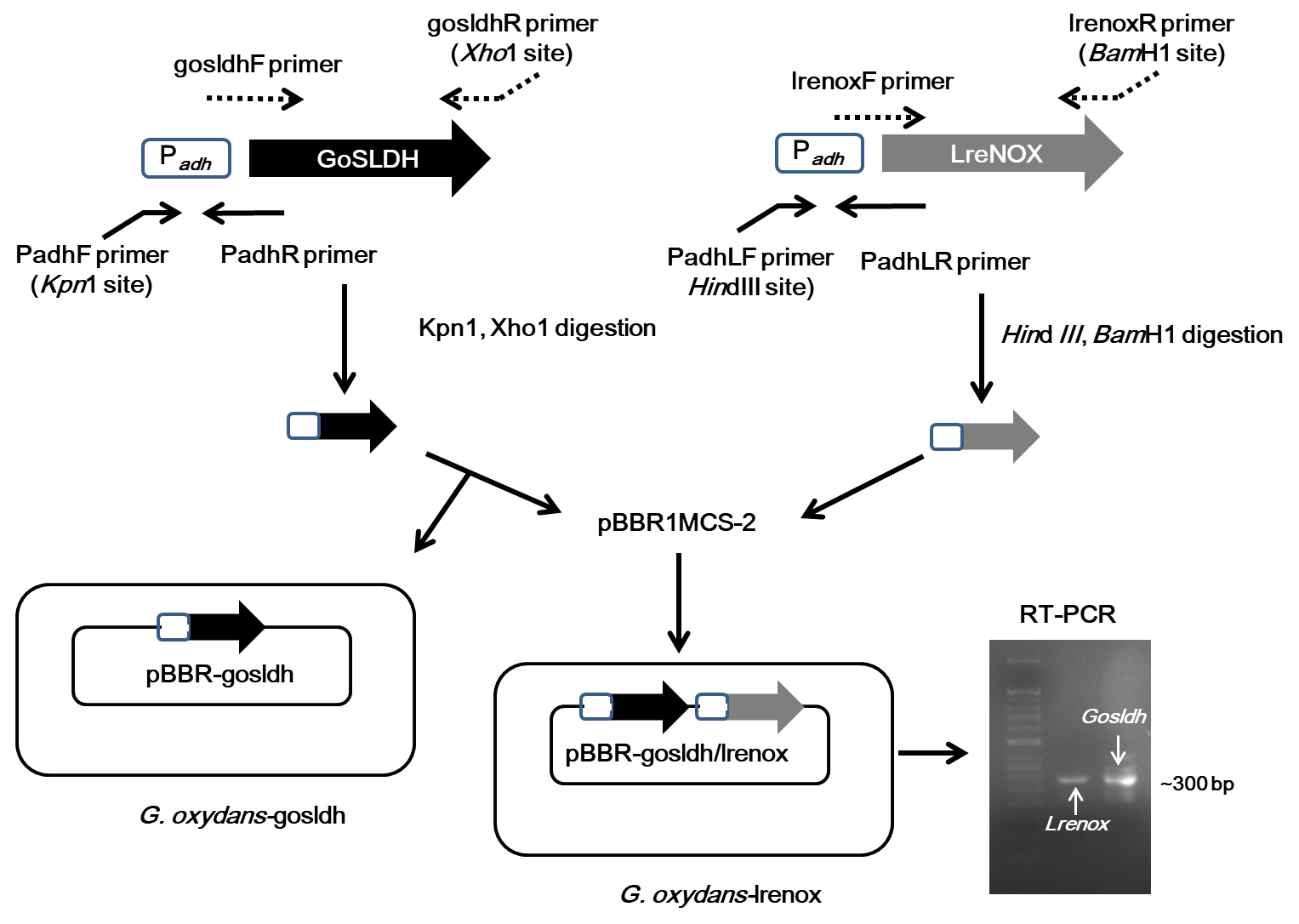


Fig. S3. Construction of *G. oxydans*-gosldh and *G. oxydans*-gosldh/lrenox. Overlap PCR was used to introduce a P*_adh_* promoter region in front of the *gosldh* and *lrenox* genes. Synthesized primers employed during this PCR are shown in Table S1. Fused *gosldh* and *lrenox* PCR product was digested using *Kpn*1/*Xho*1 and *Hind*III/*Bam*H1, respectively. The two digested PCR products (*gosldh* and *lrenox*) were then ligated into pBBR1MCS-2, which was then transformed into *G. oxydans* KCTC 1091. Gene expression of *gosldh* and *lrenox* was confirmed by RT-PCR.


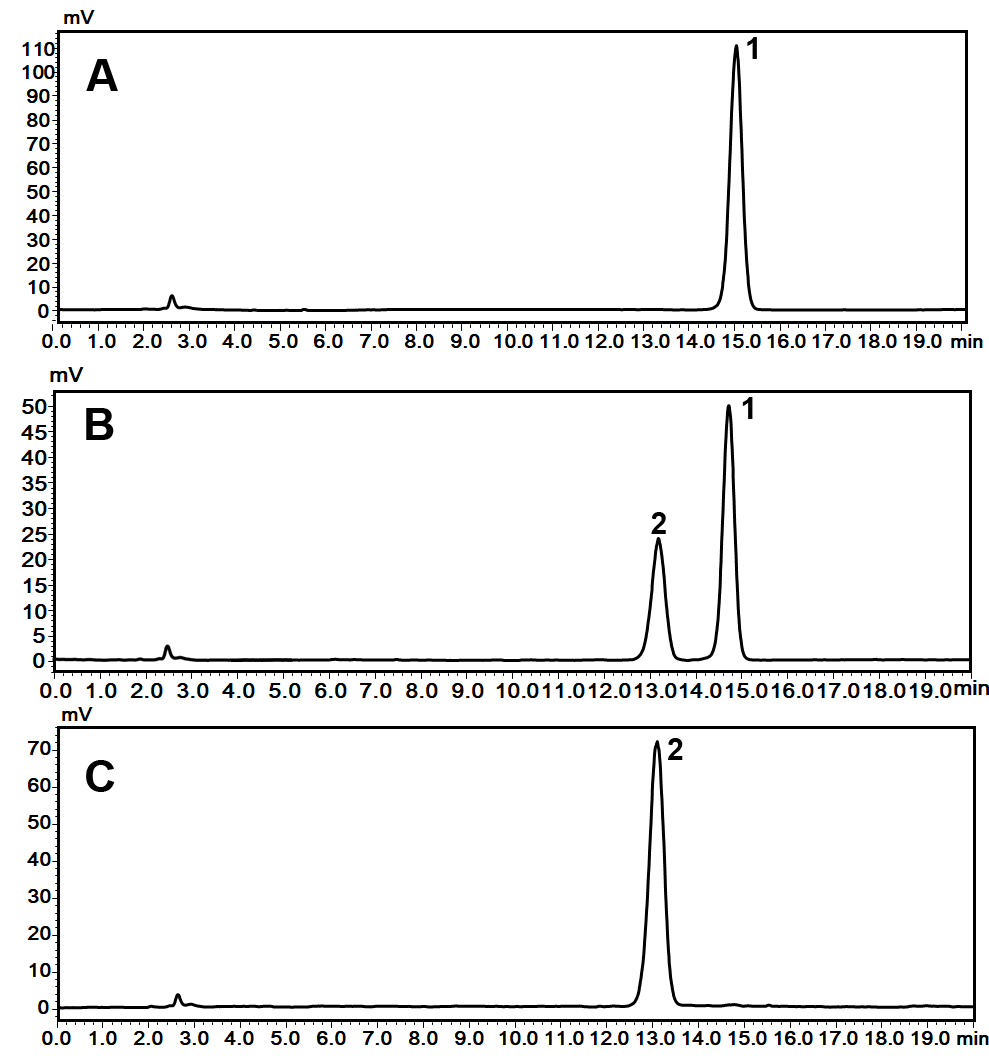


**Figure S4.** HPLC analysis of *G. oxydans*-gosldh and *G. oxydans*-gosldh/lrenox reaction product. (A) sorbitol medium, (B) *G. oxydans*-gosldh reaction product, (C) *G. oxydans*-gosldh/lrenox reaction product. Substrate (D-sorbitol) and product (L-sorbose) concentrations were estimated by HPLC using a Shodex Asahipak NH2P-50 4D (Showa Denko K. K, Tokyo, Japan) column with RI detector and using acetonitrile and water (85:15) as solvent system with a flow rate of 0.6 ml/min at room temperature. 1: D-sorbitol, 2: L-sorbose

Table S1. Primers used in RT-PCR

| Gene | Primer | Sequence (5’-3’) | Product size (bp) |
| --- | --- | --- | --- |
| *gosldh* | gosldh-F | TGCGTCGTCGTTGGGATG | 300 |
|  | gosldh-R | CATCCGCAAACTGGTCTT |  |
| *lrenox* | Lrenox-F | AACGTTGCTAAAGATGCG | 300 |
|  | Lrenox-R | GCCAACACAGAGGATAGC |  |
